# Perinatal outcomes of twenty-five human immunodeficiency virus-infected pregnant women: Hacettepe University experience

**DOI:** 10.4274/jtgga.galenos.2019.2019.0033

**Published:** 2020-09-03

**Authors:** Ahmet Çağkan İnkaya, Gökçen Örgül, Nurhayat Halis, Şehnaz Alp, Ateş Kara, Özgür Özyüncü, Murat Yurdakok, Serhat Ünal, M. Sinan Beksaç

**Affiliations:** 1 Department of Infection Diseases and Clinical Microbiology, Hacettepe University Faculty of Medicine, Ankara, Turkey; 2 Department of Obstetrics and Gynecology, Division of Perinatology, Hacettepe University Faculty of Medicine, Ankara, Turkey; 3 Department of Pediatric Infectious Diseases, Hacettepe University Faculty of Medicine, Ankara, Turkey; 4 Department of Child Health and Diseases, Hacettepe University Faculty of Medicine, Ankara, Turkey

**Keywords:** HIV, Pregnancy, antenatal care, Turkey

## Abstract

**Objective:**

To evaluate perinatal outcomes in human immunodeficiency virus (HIV) infected pregnant women in Turkey.

**Material and Methods:**

Maternal characteristics, pregnancy complications, laboratory findings including HIV load, CD4 cell count, CD4/CD8 ratio, neonatal features and final HIV status of the baby were retrospectively analyzed.

**Results:**

The sample included 26 singleton pregnancies, from 25 HIV-infected women. The ethnicities were Turkish (n=18), East European (n=4), Asian (n=2) and African (n=2). The majority (76.9%) was aware of their HIV status before becoming pregnant. Four cases (15.3%) were diagnosed during pregnancy and two (7.8%) at the onset of labor. The results for median HIV viral load, CD4 count, and CD4/CD8 ratio at birth were 20 copies/mL (0-34 587), 577/mm^3^ (115-977), and 0.7 (0.1-1.9), respectively. The HIV viral load rate was 5.5% in eighteen women taking anti-retroviral treatment. The rates of gestational diabetes mellitus, gestational hypertension, intrauterine growth restriction, and preterm delivery were 3.8%, 3.8%, 7.6%, and 8% (numbers are 1;1;2;2), respectively. The mean gestational week at birth was 38 weeks and mean birthweight is 2972±329 g. Two babies were congenitally infected with HIV (infection rate of 8.3%). There was one needle-related accident during surgery.

**Conclusion:**

Timely diagnosis of HIV infection during pregnancy is important for preventing mother to child transmission. HIV infected women may give birth to HIV negative babies with the help of a multidisciplinary team, composed of perinatology, infectious diseases, and pediatrics specialists.

## Introduction

According to estimations in 2015, 36.7 million people were infected with human immunodeficiency virus (HIV), globally (1). Among them, 17.8 and 2.1 million were “women over 15 years of age” and children, respectively. Nearly half of infected women have access to treatment, and 43% of children are receiving treatment (1). Treatment coverage for children is therefore somewhat restricted, which highlights the importance of preventive measures in early childhood, including preventing mother-to-child transmission (MTCT). The number of newly-infected children has decreased by 47%, since 2010. Maternal antiretroviral therapy (ART) is the backbone of MTCT preventive measures but, as of 2016, 24% of pregnant women in need cannot access ART (1).

Over the last 30 years, the HIV landscape was revolutionized by the advent of new class antiretrovirals (ARVs). Currently, HIV-infected people enjoy a similar life-expectancy and quality of life as their uninfected counterparts. Annually, up to 8800 (95% confidence interval 8400-8800) HIV-infected women give birth in USA (2). An orchestrated team effort is necessary for good reproductive health, family planning preconception health services, and prevention of MTCT (3).

As reported by the Turkish Ministry of Health in 2019, over 20.000 people are living with HIV infection in Turkey (4). Despite the low prevalence of HIV (<0.001%) in Turkey, the number of newly diagnosed cases increases by 452% after 2010 (5). In addition, women constitute 25% of those living with HIV or acquired immune deficiency syndrome (AIDS), known as people living with HIV/AIDS (PLWHA), in Turkey (6).

MTCT was recognized in 1982 as a mode of HIV infection and numerous prevention efforts have been investigated and reported subsequently (7). Pre-conception counselling, antenatal HIV screening, ART, and access to perinatal follow-up are key preventive measures (3,8). As a result of successful implementation, the MTCT rate in the UK decreased from 25.6% in 1993 to <0.5% in 2011, which is considered a major success (8,9). Furthermore, the ACTG076 study confirms that zidovudine (ZDV) monotherapy lowers the risk of MTCT and ART can further decrease that from 10.4% to 1.2% (10,11).

Concerns regarding the teratogenic risk of ART were alleviated by emerging data and, subsequently, maternal ART became the cornerstone of MTCT prevention strategies (12). MTCT in a non-breastfeeding setting will occur during pregnancy, which emphasizes the importance of prenatal care (8). In addition, premature rupture of membranes is associated with increased MTCT risk (13). Use of a planned caesarean section (CS) is found to lower the risk of transmission from 10.5% to 1.8% (14). In addition, maternal viral load at delivery is another major risk factor. If perinatal maternal viral load is below 50 copies/mL, MTCT risk drops below 0.5%, regardless of treatment or delivery mode (15).

Despite the growing problem of HIV infection in Turkey, there is a scarcity of real-world data from Turkish research centers. This study aimed to evaluate the Hacettepe University Hospital Antenatal Care/HIV cohort, in terms of obstetric and perinatal outcomes.

## Material and Methods

Hacettepe University Hospital is a tertiary referral center, located in the capital city of the Turkish Republic. The center provides multidisciplinary treatment for HIV-infected people, as well as for high-risk pregnancies. The Hacettepe University Hospital HIV cohort is composed of 636 PLWHA, as of October 2017, who are registered with the Infectious Diseases Clinics of Hacettepe University. Among the cohort, 92 (14.4%) are female. This study consists of all HIV-infected pregnant women who delivered at the hospital between January 2009 and October 2017. Early pregnancy losses before the 22^th^ gestational week were excluded from analysis. During the study period, 26 deliveries occurred, in 25 individual HIV-positive women.

Data was extracted from patient files, as well as from digital records. Maternal age, obstetric history such as gravida, parity, coexisting diseases, and length of hospital stay were recorded for each patient. Main pregnancy complications, such as gestational diabetes mellitus (DM), preeclampsia/gestational hypertension, preterm contractions, and premature preterm ruptures of membranes, were also noted. Laboratory findings regarding HIV virus load, CD4 cell count, CD4/CD8 ratio, and hemoglobin concentration were recorded separately during pregnancy and at birth. Details of the pregnancy follow-up protocol used are given below. Neonatal features, such as birthweight, Apgar scores at first and fifth minute, gestational week at birth, requirement for neonatal intensive care, duration of hospitalization, and final HIV status were analyzed. Antiretroviral prophylaxis with nevirpine (7 mg/kg bid or 200 mg/m^2^ bid) was administered to all babies regardless of maternal and neonatal plasma HIV-RNA result for 12 weeks. Antiretroviral treatment was commenced in all babies with positive plasma/cord blood HIV-RNA (detectable plasma HIV-RNA).

For known HIV-positive women, there is an integrated pregnancy follow-up program at the hospital center. PLWHA over eighteen years of age are routinely followed up in the infectious diseases department. All HIV-infected patients willing to conceive undergo extensive pregnancy counselling and, if special help is needed, the divisions of perinatology and andrology are involved. Upon pregnancy, the women are referred to the division of perinatology for regular follow-ups. The antenatal care program is initiated quickly after pregnancy diagnosis, to prevent MTCT for cases involving known HIV infection. The center will also accept newly diagnosed PLWHA, referred from other centers, for pregnancy follow-up. The follow-up is comprised of routine prenatal ultrasonography examinations, combined or triple-test aneuploidy screening tests, glucose challenge test between the 24^th^ to 26^th^ gestational week, and a non-stress test after the 37^th^ gestational week. Further evaluations are completed according to obstetric indications until delivery. A pediatric infectious disease consultation takes place in the last trimester, to further inform parents on postnatal management.

HIV-infected pregnant women are evaluated on admission, as recommended by international guidelines (3). All patients undergo routine testing, including complete blood count, extensive biochemical work-up, virological work-up (viral load, genotypic ART resistance testing), immunological work-up (CD4/CD8 count), and documentation of childhood immunization and diseases. ART is started before genotypic resistance testing, as recommended by international guidelines. The treatment regime is mainly composed of a Nucleoside reverse transcriptase inhibitor (NRTI) and a protease inhibitor (PI) such as boosted-lopinavir (LPV/r). PI is preferred, as pregnancy outcomes with high genetic barrier Integrase and strand transfer inhibitor (INSTI)-based regimes are obscure and INSTIs were introduced recently in Turkish market. Pregnant women are checked on a monthly basis for HIV viral load and side effects of treatment. If the maternal HIV viral load cannot be suppressed below 200 copies/mL, or viral-rebound occurs during the third trimester, the daily dose of LPV/r is increased by 50% (3x400/100 mg), without checking plasma drug levels. If an HIV-infected woman presents late in the course of pregnancy, or viral load remains high during the last trimester, intensive ART with NRTI, PI and raltegravir is given.

Caesarean section after the 38th gestational week is performed after counselling with parents. Regardless of maternal HIV viral load, a ZDV infusion is given before surgery and continued until cord-clamping. All the newborns were bathed twice and admitted to pediatric wards for close monitoring. Institutionally, breast feeding of PLWHA was clearly forbidden at all times and neonates were fed through formulary compounds. Antiretroviral prophylaxis was started with ZDV syrup. Neonates born to mothers with unsuppressed HIV viral load were further evaluated for combined antiretroviral regimes as dictated by Turkish Guidelines (16).

The study protocol was reviewed and approved by a Local Review Board (Hacettepe University Non-Interventional Clinical Studies Board decision approval number: GO-18/186-29, March 20, 2018).

### Statistical analysis

The data was analyzed using the SPSS, version 23 (IBM Inc., Armonk, NY, USA). Qualitative data is presented as percentage and frequency, whereas quantitative data is presented as mean, standard deviation, and number.

## Results

**Figure 1:**
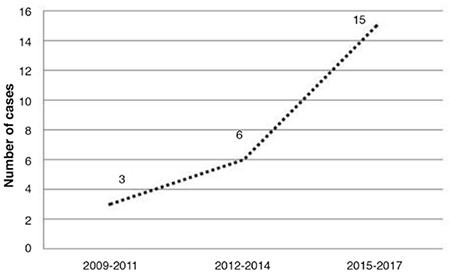
Total number of deliveries among human immunodeficiency virus infected women

During the study period, 26 singleton pregnancies in 25 HIV-infected women were recorded. Maternal characteristics and main findings of the study is given in the Table 1. The total number of cases increased from 2009 to 2017, as shown in Figure 1. Mean maternal age at birth was 27.5±6.6 years. Most participants (n=18, 72%) were of Turkish ethnicity. Of the remaining eight women, three had East European, two had Asian, and two had African origin. A previous abortion was noted in ten (38.4%) of the 26 women, six of whom had a single abortion, three miscarried twice, and one had three previous abortions. Overall, there were twelve primigravid women (46.2%) in the study group.

Six coexisting diseases were noted in the 25 women, including chronic Hepatitis C, major depressive disorder, asthma and tuberculous empyema (n=2;2;1;1). Twenty women (76.9%) knew their HIV status before becoming pregnant and eighteen were already on ART. Two HIV-positive patients refused to take medications during pregnancies. Two women (15.3%) were diagnosed during pregnancy and the remaining two (7.8%) were diagnosed at the onset of labor. ARV was given to the latter four cases immediately after diagnosis. Of the two women presenting at delivery, one delivered by CS under ZDV prophylaxis and the last one did not receive perinatal prophylaxis, due to late admission and urgent CS.

Laboratory test results were collected for the twenty participants. We analyzed the laboratory parameters for all the women, except the two diagnosed at birth. The median HIV viral load, CD4 count, and CD4/CD8 ratio during the first trimester were 2203 copies/mL (0-529,000), 460/mm^3^ (26-786), and 0.7 (0.04-1.3), respectively. The results for median HIV viral load, CD4 count, and CD4/CD8 ratio are 20 copies/mL (0-34 587), 577/mm^3^ (115-977), and 0.7 (0.1-1.9), respectively in the last trimester.

The blood HIV viral load was under 200 copies/mL at birth in the twenty pregnant women. After excluding the two participants with missing data, four women (16.7%) were found to have an HIV viral load below 20 copies/mL before delivery. Within the study sample, two refused treatment, one was receiving ART, and one had a late diagnosis. The positive HIV viral load rate was 5.5% in the eighteen women receiving ART. There were 23 participants with full data on CD4 count and CD4/CD8 ratio. When participants were classified into groups, according to their CD4 count, seventeen were below 500/mm^3^, two were between 350-500/mm^3^, two were between 200-350/mm^3^, and two were above 200/mm^3^. The women were further divided in terms of CD4/CD8 ratio. In regard of CD4/CD8 ratio, eight were above 1, nine were between 0.5-1, three were between 0.2-0.5, and three were below 0.2.

There were three PLWHA with viral load count below 200 copies/mL. Two of three were late presenters, they presented at term and thus they did not receive any prophylactic treatment. The third woman was on trimethoprim/sulfamethoxazole (1 ds tablet/day) prophylaxis during gestation. However, it was discontinued after pregnancy was diagnosed. Immunization of the PLWHA is of concern. All the patients applying to our center has been evaluated for childhood vaccinations and respective serology results. Immunization included conjugated *pneumococcus*, polysaccharide pneumococcus, seasonal influenza, diphtheria-tetanus-acellular pertussis, mumps-measles-rubella, varicella zoster, hepatitis-B and hepatitis-A vaccines. Live cell vaccines were contraindicated in pregnant woman. All vaccinations were performed according to Turkish HIV treatment guidelines (16).

After excluding data from one participant with late admission and missing data, the remaining 25 women were evaluated for pregnancy-related complications. Three instances of hospitalization during pregnancy were noted (two with pneumonia and one with gastroenteritis). One woman (3.8%) developed insulin-dependent gestational DM. Additionally, gestational hypertension was present in one (3.8%) woman.

We did not observe a premature rupture of membranes among any of the participants. Four women were hospitalized, due to preterm contractions, and two of those women delivered before the 37th gestational week. As a result, the preterm delivery rate was 8%. Also, intrauterine growth restriction was recorded in only two cases (7.6%).

There were eleven male (42.3%) and fourteen female (53.8%) babies born to the study sample. The mean gestational week at birth was 38 weeks, with a range from 35 0/7 to 40 1/7 weeks. There were two (7.7%) vaginal deliveries within the group and the CS rate was 92.3%. Mean birthweight was 2972±329 g. Mean Apgar scores at first and fifth minute were 8.4 and 9.4, respectively. Neonatal resuscitation in the delivery room was performed for one infant after birth. Furthermore, the HIV status of two babies was not available from patient files. Of the remaining 24, only two were HIV-infected, showing a low MTCT rate of 8.3%. There were no stillbirths, perinatal mortalities, or congenital abnormalities.

One HIV-infected baby was born to a 39 year-old mother, gravida 2 and parity 1, whose HIV status was first detected within the first trimester. The expectant woman had a high viral load (256.000) at the time of diagnosis and was given treatment immediately. The baby’s HIV-RNA was 56, CD4 was 266, and CD4/CD8 ratio was 0.4 at birth. The baby was delivered via CS in the 38^th^ gestational week and weighed 3230 g. Records for the other HIV-infected baby show the mother was diagnosed with HIV at delivery. The maternal laboratory findings were HIV-RNA 11.100 copies/mL, CD4 count 562/mm^3^, and CD4/CD8 ratio was 0.8. The CS delivery was performed in the 38^th^ gestational week and the 3,470 g fetus was transferred to the neonatal intensive care unit.

Mean hemoglobin decrease after delivery was 1.7±0.9 and there was no need for red blood transfusion for either infant. A surgical site infection developed in one mother (3.8%), which was treated with empiric antibiotics and wound care. There was one needle-stick injury which occurred during delivery, affecting a member of the surgical team, but antiretroviral prophylaxis was deferred as the index-patient had undetectable HIV-RNA.

## Discussion

Despite decreasing trends across the world, HIV infection incidence is increasing in Turkey, due to a lack of knowledge and stigma (4,5). Effective interventions must include multidisciplinary teams and involvement of relevant stake holders. This, in turn, necessitates increasing scientific information available at every level. In this study, the obstetric and perinatal outcomes of HIV-infected woman were evaluated in the Hacettepe University Hospital cohort. This is the first scientific report on obstetric outcomes in the HIV-infected Turkish population.

In accordance with increasing HIV incidence in Turkey, the number of HIV-positive pregnancies is also increasing. This may be due to changes in epidemiology and understanding of HIV infection among the population at risk (17). There was a near five-fold increase in HIV-positive pregnancy rates over the last three years.

Contrary to previous reports, our study records intentional pregnancies among the women receiving ART. Unintended pregnancies risk harm to the mother and to the baby. Furthermore, unintended pregnancies among HIV-infected women are associated with delayed antenatal care, poor fetal outcomes, and poor retention of postpartum care (18,19).

HIV-infected sero-discordant or sero-concordant partners are recommended to receive reproductive counselling before conception, including identification of coexisting conditions and risk factors associated with adverse maternal and fetal outcomes (20). Ideally, all sexually active women require screening for HIV infection before considering pregnancy (3). Raffe et al. (8) report 72% of pregnant women know their HIV status before conceiving. Furthermore, in a study of a large British cohort, antenatal HIV serostatus awareness was shown to increase from 24.6% between 2000 and 2006 to 12.5% between 2007 and 2011 (9). The Republic of Turkey Ministry of Health recommends an HIV screening test with the consent of the pregnant woman in new prenatal care management guidelines in 2018 (21). Most centers, though, test all pregnant women in their first trimester and at birth. Our results show the importance of HIV screening during pregnancy in Turkey, as 15.3% of participants are diagnosed during pregnancy and 7.8% are diagnosed at onset of labor.

Risk of MTCT may be affected by many factors, including high maternal viral load, lower CD4 count of the mother, mother with AIDS defining disease, premature rupture of the amniotic membrane, preterm delivery and breastfeeding (22). As maternal viral load is the main determinant of these risk factors, ART delivered during pregnancy is the cornerstone of MTCT elimination strategies. Recent guidelines recommend the use of ARTs, regardless of viral load and immunologic status, as the preventative effect is present irrespective of these factors. Furthermore, ART drugs can affect the fetus through the placenta and act as pre-exposure prophylaxis (23). Initiation of PI-based ART is associated with preterm deliveries in univariate analysis but not multivariate analyses (24). The main goal of ART during pregnancy is to suppress the virus to an undetectable level at the time of delivery so that MTCT will be an infrequent event. Maintaining a pregnancy-compatible ART is recommended in all women. Women conceiving under ART should be evaluated for possible adverse outcomes of certain antiretroviral drugs. Dolutegravir has been associated with neural tube defects (NTD) in the newborn. However, recent data shows decreased NTD risk when compared to previous report (25,26). However, the debate over antiretroviral associated NTD continues, so that drugs with a better safety profile should be preferred during pregnancy. These include LPV, raltegravir and efavirenz (especially in resource poor settings). In our cohort two MTCT events were seen. The underlying reason for transmission was late diagnosis in one case and high viral load at first trimester and delivery in the second case. High viral load at birth is related with an increased risk of MTCT according to our findings, which is consistent with existing literature (2,9,17).

PI-based treatments, including ritonavir, have been associated with preterm delivery (3). The potential mechanism underlying this effect remains unknown. Our results demonstrate that preterm delivery rate with PI-based treatments was 8%. Despite preterm delivery risk, PI-based ART promises various advantages compared to other regimes. In a resource-poor setting, with little access to genotypic resistance testing, the high genetic barrier of PIs provides an opportunity to administer ART without genotypic resistance test results. Moreover, PIs are potent drugs and lower viral load extremely rapidly (27). As distribution volumes may change during the third trimester, LPV levels are usually checked beforehand (28). We did not have access to therapeutic drug monitoring for ARTs. Therefore, the pregnant women were closely monitored for HIV viral load during the third trimester. In a case of virologic rebound during the third trimester, daily dosing would be increased by 50%, as recommended by Manavi et al. (29).

In addition to the importance of MTCT, HIV-positive pregnancies are vulnerable to several other complications. A previous study showed no increased risk for preeclampsia, preterm birth, or smallness for gestational age, in women receiving treatment (30). A more recent meta-analysis demonstrated a two-fold increase in risk for preterm delivery and low birthweight in HIV-positive pregnancies (31). Our results show a preterm delivery rate of 8% and Intrauterine growth restriction rate of 7.2%. The number of cases is low in this study, so it is not possible to calculate a definitive frequency of prematurity among these cases.

The mode of delivery is dependent on multiple factors in HIV infected pregnant women. Previously, elective CS was recommended to minimize the risk of MTCT in all these pregnancies (15). With the findings of recent studies, vaginal delivery is shown to be safe for neonates if maternal viral load is <1,000 copies/mL. Thus, CS should be performed only for obstetric indications such as placenta previa, previous CS history, malpresentation, fetal distress, and the like (32). The high rate of CS in our cohort indicated that clinicians tended to choose elective CS, most probably due to medicolegal issues.

Pregnancy-induced hypertension and preeclampsia are important causes of maternal morbidity and mortality. Fortunately, studies have reported the risk of preeclampsia was not increasing in HIV-infected women (33,34). We also showed that the frequency of preeclampsia was only 3.8%. GDM is another concern for HIV-positive pregnant women, due to related medication and infection. Previous studies, however, reported the risk of GDM was not increasing in HIV-positive expectant women, as compared to healthy pregnancies (35,36). Our findings are in concordance with the relevant literature.

### Study Limitations

The major limitation of the current study is the small number of cases and the single center nature of the cohort. In addition, missing and unreliable data due to the retrospective design of the study was a further limitation.

## Conclusion

Timely diagnosis of HIV infection during pregnancy is important for preventing MTCT. HIV infected mothers may give birth to HIV negative babies with the help of multidisciplinary teams, composed of specialists in perinatology, infectious diseases, and pediatrics.

## Figures and Tables

**Table 1:**
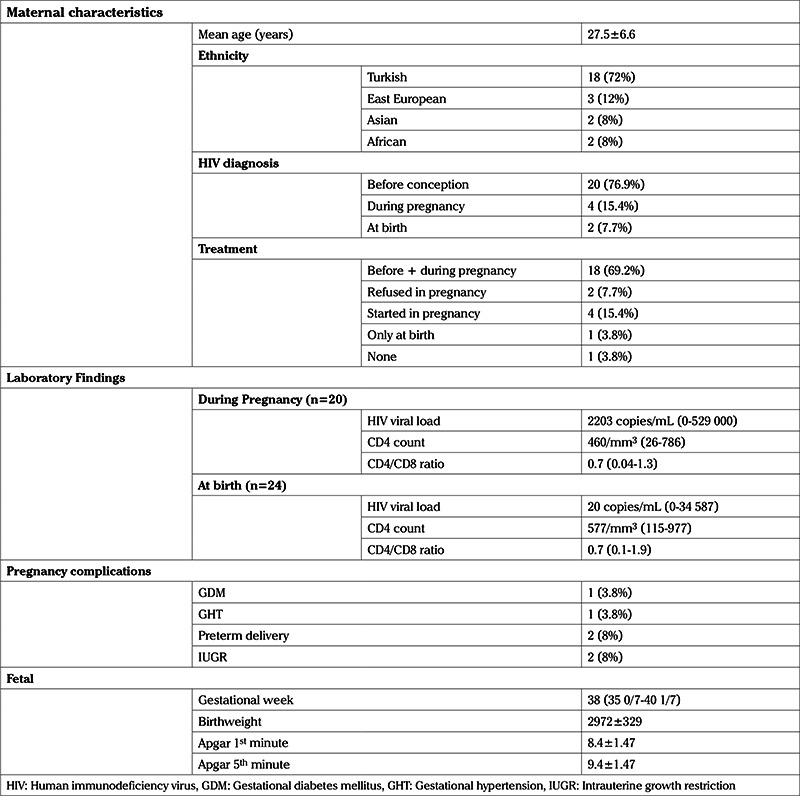
Maternal characteristics and main findings
